# Mutants in the Mouse NuRD/Mi2 Component P66α Are Embryonic Lethal

**DOI:** 10.1371/journal.pone.0000519

**Published:** 2007-06-13

**Authors:** Susan Marino, Roel Nusse

**Affiliations:** Howard Hughes Medical Institute, Department of Developmental Biology, Beckman Center, Stanford University School of Medicine, Stanford, California, United States of America; Fred Hutchinson Cancer Research Center, United States of America

## Abstract

**Background:**

The NuRD/Mi2 chromatin complex is involved in histone modifications and contains a large number of subunits, including the p66 protein. There are two mouse and human p66 paralogs, p66α and p66β. The functions of these genes are not clear, in part because there are no mutants available, except in invertebrate model systems.

**Methodology:**

We made loss of function mutants in the mouse p66α gene (mp66α, official name Gatad2a, MGI:2384585). We found that mp66α is essential for development, as mutant embryos die around day 10 of embryogenesis. The gene is not required for normal blastocyst development or for implantation. The phenotype of mutant embryos and the pattern of gene expression in mutants are consistent with a role of mp66α in gene silencing.

**Conclusion:**

mp66α is an essential gene, required for early mouse development. The lethal phenotype supports a role in execution of methylated DNA silencing.

## Introduction

**Figure 1:**
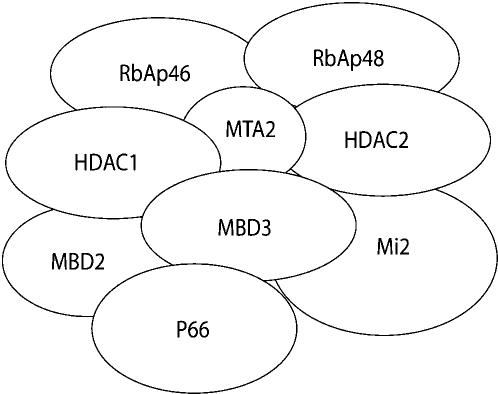
Composition of the NuRD/Mi2 chromatin complex, including p66. Together with NuRD/Mi2 and MBD2 , p66 comprises the MeCP1 complex. MBD2 and MeCP2 can recruit histone deacetylase activity (HDAC) to methylated DNA See the text for references.

Epigenetic changes in gene expression, such as occurring during imprinting and X chromosome inactivation, can result from DNA methylation and from chromatin remodeling. The latter includes histone modifications such as deacetylation [1], acetylation [2], and methylation [3]. Histone modifications and DNA methylation are functionally linked to each other. Histone modifications are catalyzed by a number of enzyme complexes, including the NuRD/Mi2 complex [4]. DNA methyltransferases maintain previously existing methylation (Dnmt1) and apply methyl groups to previously non-methylated sites (Dnmt3a and Dnmt3b) [5], [6]. Proteins that bind preferentially to methylated DNA have been isolated. These include MeCP2, and MBDs 1, 2, and 4 [7]–[9]. Both MBD2 [10] and MeCP2 [11] can recruit histone deacetylase activity to methylated DNA, and this activity has been shown to repress reporter genes in cell culture studies. MBD2 interacts with the NuRD/Mi2 complex (Figure 1) and acts in the nucleus where it is localized to both euchromatic and heterochromatic foci, suggesting a role in facultative gene activity [10]. MeCP2 interacts with the Sin3A complex and localizes primarily to pericentric heterochromatin [12], [13], indicating that it may contribute to chromosome stability.

Another protein in the NuRD/Mi2 chromatin complex is termed p66, cloned in *Drosophila* as a genetic modifier of Wingless signaling [14]. Together with NuRD/Mi2 and MBD2, p66 comprises the MeCP1 complex [15], [16] (Figure 1). There are two human p66 paralogs, p66α and p66β [17], encoding proteins with two conserved regions known as CR1 and CR2. The CR1 domain has been shown to bind strongly to MBD3, a scaffolding protein of the NuRD/Mi2 complex [17]. The 3′ conserved region of p66α, the Zn finger-containing CR2 domain, also interacts with MBD3, while the CR2 region of p66β targets the complex to multiple individual chromosomal loci in cell culture experiments [17]–[19].

To characterize the activity of p66 and assess its importance in mouse development, we have made loss of function mutants in the mouse p66α gene (mp66α, official name Gatad2a, MGI:2384585). We report here that the gene is required for normal embryogenesis.

## Results

### Cloning of mp66α, Genomic map, restriction mapping, and knock-out strategy

**Figure 2:**
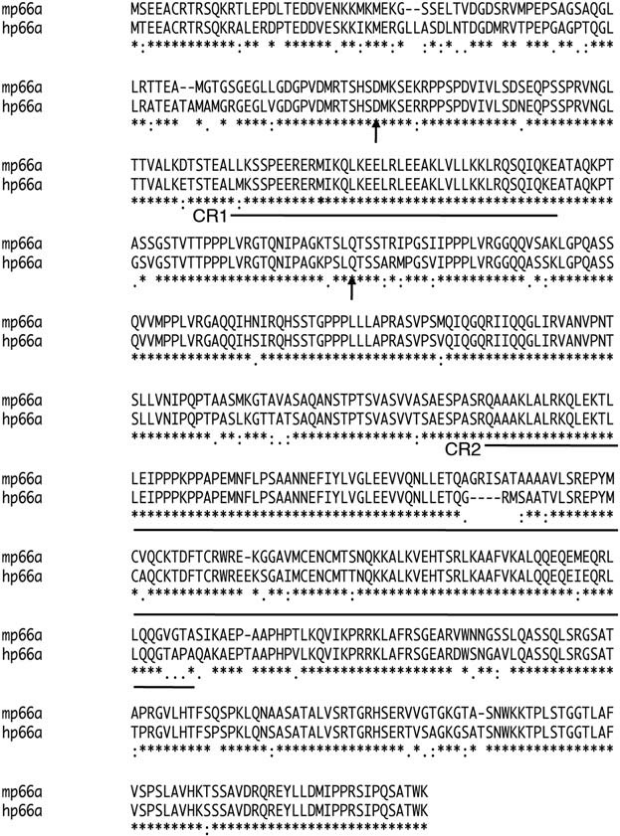
Sequence alignment of mouse and human p66α proteins. Identical residues are indicated by a *. The coiled-coil (CR1) and GATA type zinc finger (CR2) regions are underlined. The arrows indicate the positions of the boundaries of Exons 2 and 4, which are eliminated after gene targeting. The truncated protein consists of the region upstream from the first arrow, followed by 34 frameshifted amino acids.

**Figure 3:**
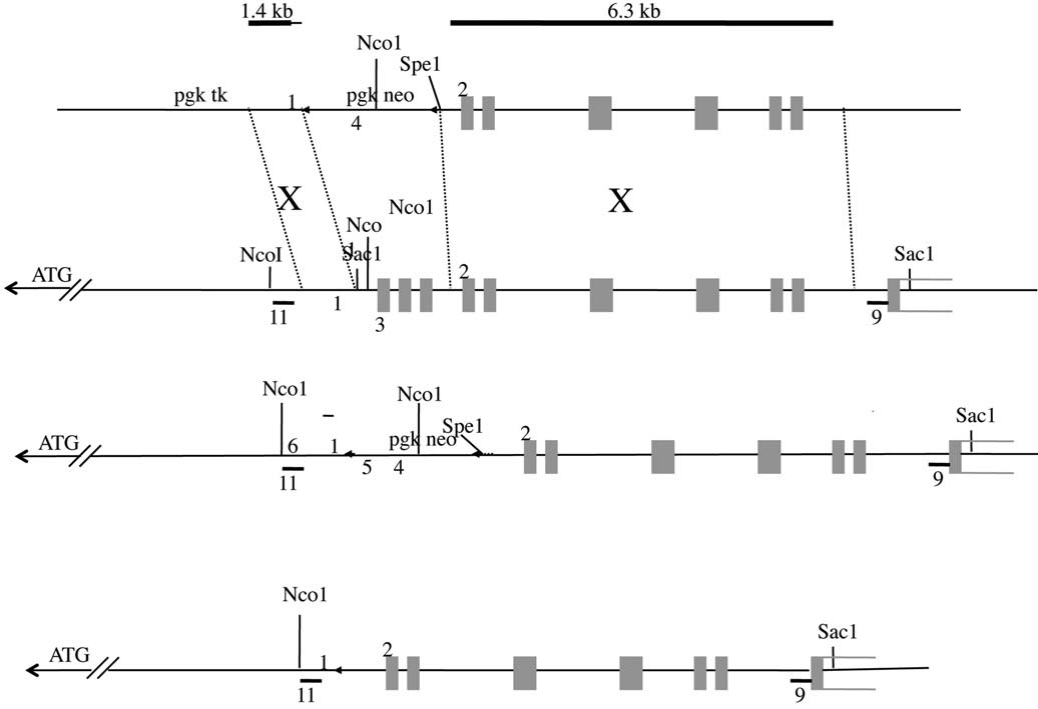
mp66α Knock-out and Genotyping Strategy. A: ploxPNT mp66α targeting vector. B: wild type mp66α genomic region. C: mutant mp66α genomic region. D: mutant mp66α with pgkneo cassette removed. Gray rectangles are exons. Heavy black lines at top indicate location of homologous arms. 5 and 6 are primers N-6 and 2-36 respectively. They were used to screen targeted clones by long range PCR for homologous recombination. The expected band size was 2069 nt. Labeled restriction sites and external probes 9 and 11 were used to confirm correct targeting and for some genotyping. 1–4 are primers used for PCR genotyping after recombination was shown to be correct by Southern blot genotyping. Short Arm: Probe 11 Long Arm: Probe 9 NcoI WT = 2335 bp Spe I, Sac I WT = 8724 bp NcoI mutant = 2799 bp Spe I, Sac I mutant = 7633 bp

By a combination of EST and cDNA mapping, we assembled a 6.4 kb cDNA contig for mp66α. The transcript theoretically encodes a protein of 630 amino acids with 87% identity and 93% identity or similarity with human p66α (Figure 2). The protein consists of two known conserved regions (CR): CR1 contains a coiled-coil, a known protein-protein interaction domain, and CR2 possesses a GATA type zinc finger region (Figure 2). To design a construct for a targeted deletion, we isolated several BAC clones from mouse strain 129/Sv and mapped the location of the introns. The gene consists of 14 exons distributed over 15 kilobases (Figure 3). We developed a strategy of homologous recombination replacing the exons encoding CR1 and the two flanking exons (exons 2–4) by a floxed pgk-neo cassette (Figure 3). The resulting protein would consist of 86 amino acids from the non-conserved region, followed by 34 amino acids not encoded by the gene (due to frame-shifting) and a stop codon well before the CR1 region (see Materials and Methods).

### Homologous recombination and generation of mp66α +/− mice

**Figure 4:**
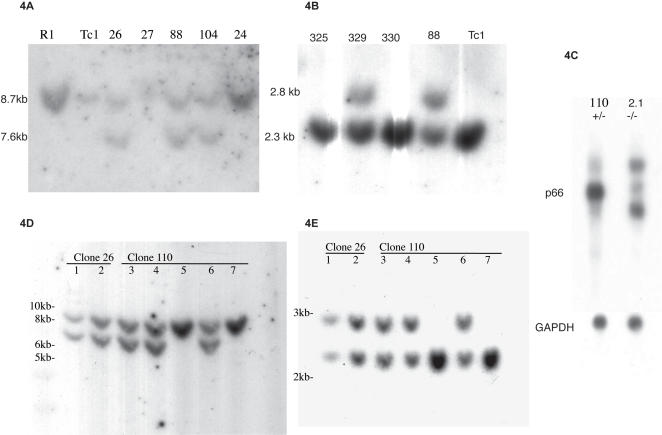
Confirmation of correct targeting by molecular hybridization. A: Southern blot of SpeI, SacI digested clones hybridized with probe 9. The long homologous arm is correctly integrated in clones numbered 26, 88,104 as indicated by the 7.6 kb fragment. B: Southern blot of Nco I digested clones hybridized with probe 11. The short homologous arm is integrated correctly in clones numbered 329 and 88, as indicated by the 2.8 kb fragment. C. Northern blot of RNA in +/− and −/− ES cells. mp66α RNA is normal in the +/− clone but aberrant in the −/− clone. GAPDH is the loading control. D, E. Pups 1, 2, 3, 4, and 6 are heterozygous for the correctly integrated mutant alleles as shown for both the long arm (D) and the short arm (E). These pups were the founders for two independently targeted lines, line 26 and line 110.

We confirmed homologous recombination in 11% of the neomycin-resistant R1 embryonic stem cells (Figure 4A, B). Northern analysis of mutant ES cells showed that the mp66α RNA was aberrant compared to wild type (Figure 4C), confirming a successful deletion. Four independent ES cell clones were injected into a total of 90 blastocysts, producing 33 pups altogether. Of these, 14 were chimeric according to coat color. Two males, one each from clones 26 and 110, produced heterozygous offspring (Figure 4D, E). Heterozygotes were viable, normal looking, and fertile, producing offspring in normal Mendelian ratios when crossed with wild type animals. Genotyping of 375 3-week-old pups from heterozygote intercrosses revealed the presence of wild-type and heterozygous animals at the expected 1∶2 ratio (Table 1), but no mp66α homozygous mutant pups were found among these animals, suggesting that the gene is essential for development.

**Table 1:** Intercross Genotypic Ratios.

Age genotype	+/+	+/−	−/−	?
newborns	123 (33%)	252 (67%)	0	0
e8.5	2	3	3	1
e9.5	11	18	6	2
e10.5	7	18	8	3
Total embryos	20 (24%)	39 (48%)	17 (21%)	6 (7%)

### Phenotypes of intercross embryos at day e8.5–e10.5

**Figure 5:**
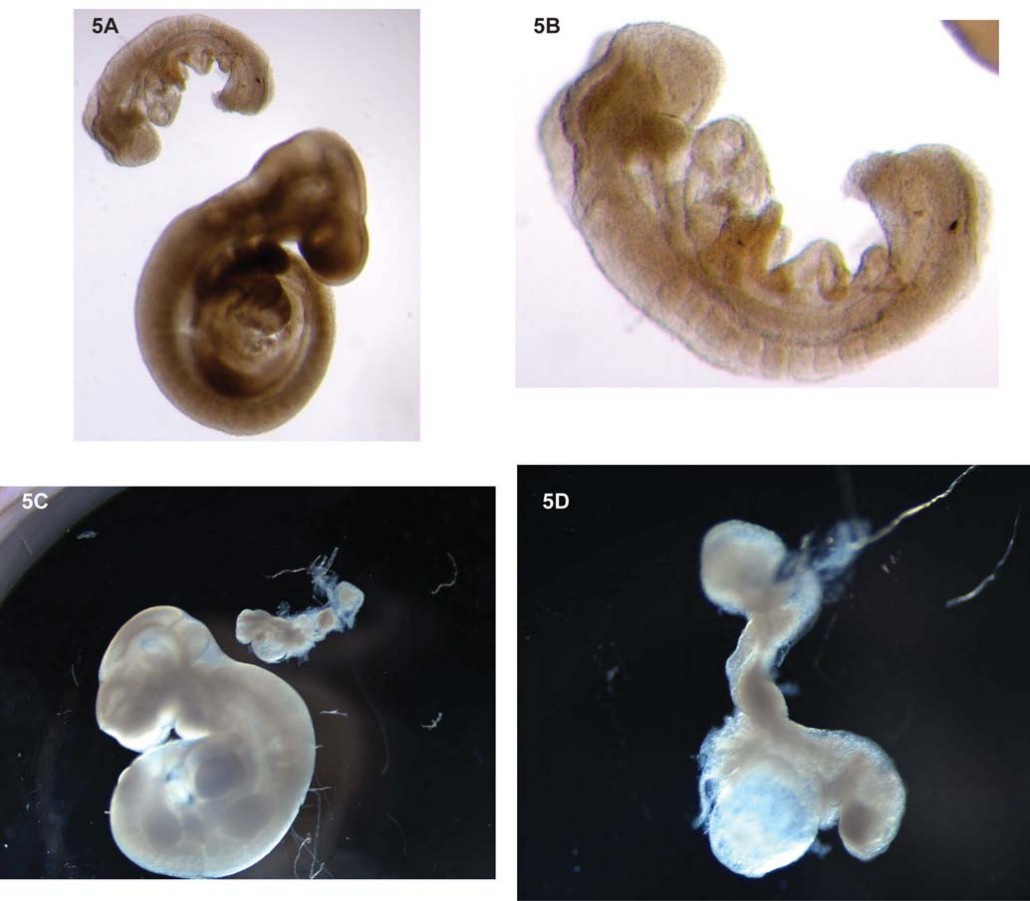
Mutant embryos. Embryonic Day 9.5 and 10.5 mp66α −/− Embryos. Mutant embryos, significantly smaller, are shown in comparison with wild-type littermates. Abnormalities in mp66α null embryos around day 9.5 (A, B) include malformed and non-fused neural folds, failure of closure of anterior neuropore, missing or excessively large blood vessels in the yolk sac, Day 10.5 embryos (C,D) are disintegrating due to multiple defects.

We examined intercross embryos at days e8.5 (embryogenesis 8.5) through e12.5. Overall, we found a wide variety of defects. Virtually every organ of candidate mutant embryos could be affected, resulting in dramatically smaller embryos and advanced stages of necrosis. We could not identify particular defects or abnormalities that would suggest a specific step in embryogenesis being dependent on mp66α. We present this analysis in tables 1 and 2, related to the timing of developmental arrest; and in figure 5 illustrating the degree of failure of development.

**Table 2:** Intercross phenotypic ratios.

Age	Total embryos	normal	arrested	Not evaluated
e8.5	40	40 (100%)	-	-
e9.5	65	51 (78%)	12 (19%)	2 (3%)
e10.5	49	34 (69%)	15 (31%)	-
e12.5	10	6 (60%)	-	4 (40%)

We found that by day e12.5, 61% of embryos were either wild-type or heterozygous, while 39% were in such an advanced stage of necrosis that they could not be examined or genotyped. For embryonic days 8.5–10.5, we initially genotyped 82 whole animals using a Southern blot protocol to account for all animals in a litter. We obtained ratios close to normal Mendelian ratios: +/+, 0.96; +/−, 1.92; −/−,0.84 (Table 1). However, of the 6 animals not genotyped in this group, 4 were less than half the size of siblings and were probably null. This would change the ratios to .96∶1.92∶1.04, indicating that all null embryos had implanted successfully. When 114 e9.5 and e10.5 animals were visually examined (Table 2), 24% displayed the arrested phenotype, supporting this finding. Therefore, mp66α is essential for development but not required for normal blastocyst development and implantation.

An examination of 83 homozygous null e7.5–e10.5 embryos from additional litters revealed a range of defects, delayed development, and much smaller size than litter mates (Figure 5), but with healthy placentas and often healthy tissue despite severe delays.

A normal day 8.5 embryo has 8–12 somites, is in the process of turning, has a paired heart primordial fusing anteriorly, and has closed neural folds at somites 4–5 [20]. Of the 27 e8.5 null embryos examined, 7 were in the egg cylinder stage as expected at day 6 to 6.5. Two of these were lumpy and abnormally shaped and two were in the early neural fold stage but were misshapen (not shown). One of these had developed a single anterior neural fold, but no somites. The remaining 18 embryos had a normal appearance with somite numbers ranging from 3 to 9 (Figure 5).

Abnormalities in mp66α null embryos around day 9.5 included malformed neural folds that were ruffled and not fused, failure of closure of anterior neuropore, missing or excessively large blood vessels in the yolk sac, turned but small underdeveloped embryos, and embryos that failed to turn despite having 20 or more somites (Figure 5).

### Differential gene expression in mp66α mutant embryos

Given the evidence that p66 is part of a repressor complex, we were interested in asking whether specific genes are affected by the absence of mp66α in embryos. We carried out an Affymetrix microarray analysis of 4 pairs of null vs. wild type day 8.5 siblings that had achieved an equal level of development as judged during dissection.

Over 45 genes were identified as being increased in the null embryos of at least three but usually all four matched sibling pairs (Table 3). Four of these genes, Psx2, Psx1(Placenta specific homeobox 2 and 1), Cx31 (Connexin 31, specific to trophoblast stem cells), and an unknown bHLH protein similar to Mash 2 are active in the trophoblast lineage in the extraembryonic tissues of the mouse. The remaining genes are either unknown, or not confined to the extraembryonic lineages. Of particular interest are two genes normally expressed in males only, but not found on the Y chromosome, Amhr2 (anti-Mullerian hormone type 2 receptor) and Ldh3 (lactate dehydrogenase 3, C chain, expressed normally in the germinal epithelium of the testis). Despite the fact that three of the null embryos were females, these genes were expressed at heightened levels.

**Table 3:** Genes differentially expressed in mp66α mutant embryos.

A.	B.	C.	D.
3.6	1425035	DNMT3l	DNA methyl-transferase 3-like
3.4 *	1432018	Mash 2	Achaete-Scute homolog-2
3.2	1433465	Unknown EST	
3.2 *X	1449540	Psx 2	Placenta specific homeobox 2
2.9	1457021	Amhr2	Anti-Mullerian hormone receptor
2.8	1417945	Pou5f1	unknown transcription factor
2.7	1416490	Tmed6	possible transmembrane protein
2.7	1415846	Ldh3	lactate dehydrogenase 3, C chain
2.5 X	1419229	E hox repeat	ES cell derived homeobox gene
2.4 *X	1419018	Psx 1	Placenta specific homeobox 1
2.2 X	1444038	unknown EST	
2.1	1417426	Sgc, Prg1	Proteoglycan 1 granules
2.1 *	1416715	Cx31, Gjb3	Connexin 31
2.0	1422943	Hspb1	Heat shock protein 1
2.0	1416023	Fabp3	Fatty acid binding protein 3
2.0 X	1417216	Pim 2	proviral integration site 2

A. Average differential expression of genes, based on four comparison numbers for the sibling pairs

B. Affymetrix code number for the sequence

C. Gene symbol

D. Gene description

* - expressed in extra-embryonic tissues

X – on the X chromosome

Because of the presumed role of p66 proteins in epigenetic gene repression, we compared the mp66α null embryonic phenotype with published mutants in other genes implicated in this process: the DNA methyltransferases, Dnmt1, the maintenance methyltransferase, and Dnmt3a and Dnmt 3b, the de novo methyltransferases, and the histone methyltransferases G9a and SUV39H [3]. G9a [21] is the histone methyltransferase thought to be responsible for H3-K9 methylation in euchromatin and therefore may be involved in developmental gene regulation. SUV39H1 [22] is the name for both SUV39h homologs of this histone methyltransferase known to be active in the pericentric heterochromatin and required for genome stability.

The mp66α null embryos were very similar to the Dnmt1 null, Dnmt3a/3b double null, and the G9a null embryos. The Dnmt1 null embryo [23] is distinguishable from wild type embryos at day e8.5 and by day e9.5 often has distorted neural tubes and no somites. The most advanced null embryos had no more than 8 somites. The Dnmt3a/3b double null embryos were of smaller size, had abnormal morphology at eE8.5 and e9.5, and died before e11.5. They had no somites and did not turn [6]. The G9a null embryos do not develop beyond the 5–6 somite stage, representative of approximately day 8.25, though they survive until day 9.5 when they begin to die. Like mp66α null embryos, some G9a null embryos arrest as early as the egg cylinder and neural plate stage [21]. The mp66α null phenotype was slightly less severe than each of these – while the embryos died at about the same time, the mp66α null embryos often had 20–30 somites. Nevertheless, the consistency of these phenotypes implicates mp66α in the epigenetic events that become important as differentiation begins.

## Discussion

The NuRD/Mi2 complex, which includes p66 is found in all fetal and adult tissues and its components are expressed throughout early embryonic development from at least the 8–16 cell stage onward [24]. Its nucleosomal remodeling ability is the highest of the SWI2/SNF2 type ATPases [25] and it is the most active HDAC activity found in *Xenopus* and mammalian cells. By making mice mutant for mp66α, we show that the gene is essential for normal development.

### The lethal phenotype is consistent with a role in execution of methylated DNA silencing

The mp66α−/− mutation results in an embryonic lethal phenotype at around day e9.5. Because methylated DNA binding proteins or activities are capable of repressing reporter genes by recruiting histone deacetylase activity, the loss of HDAC due to loss of mp66α should result in a DNA methyltransferase knockout phenotype. The maintenance methyltransferase (Dnmt1)−/− embryo dies by the eight somite stage, around day e8.5), while the de novo Dnmt3a/3b methyltransferase double null embryo dies before e11.5, with a variety of developmental abnormalities [5], [6]. This is consistent with the fact that methylation and developmental gene silencing begins in earnest when the embryo implants and gastrulation begins. The death of the mp66α−/− embryo at e9.5 with variable developmental defects supports therefore a role for mp66α in gene silencing.

The mp66α null phenotype appears to be less severe than the knock-out phenotypes of two individual NuRD/Mi2 components. The MBD3 null embryo is less developed than Dnmt−/− embryo and the HDAC1null embryo dies prior to e9.5 with a cell proliferation defect [26], [27]. Moreover, MBD3-deficient embryonic stem cells, while able to initiate differentiation in embryoid bodies, fail to commit to developmental lineages [28], whereas we found that mp66α homozygous mutant ES cells differentiate normally (data not shown). These differences may be the result of non-MeCP1 functions of the NuRD/Mi2 complex. This may not be unexpected, as mammalian p66α has only been associated with NuRD/Mi2 in the MeCP1 context, yet MeCP1 contains only about 10% of the NuRD in the cell [16].

On the other hand, we found that the mp66α phenotype is also not as severe as that due to loss of all methylation. mp66α−/− embryonic stem cells were capable of differentiating into embryoid bodies at normal rates (data not shown), while Dnmt1 homozygous mutant ES cells are reported to undergo more limited differentiation at slower rates [23]. This suggests that if the phenotype is a result of the elimination of MeCP1activity, then MeCP1 probably mediates only a subset of the methylation-dependent repression. This might be expected because it is known that the MeCP2 and the Sin3A HDAC complex are active in the pericentromeric heterochromatin [9], [29]. Alternatively, this result could mean that loss of mp66α only partially disables the MeCP1 function.

If DNA methylation were dependent upon H3-K9 methylation, and if mp66α were required for H3-K9 deacetylation, one would expect to see loss of DNA methylation in mp66α−/− ES cells, even in a hypomethylated state. We used probe for minor satellite methylation and an IAP probe to assess methylation of dispersed repetitive elements. DNA methylation at all these loci was normal in the ES cell cultures (data not shown).

Given the fact that methylated DNA binding activities and proteins have been shown to repress reporter genes through histone deacetylase activity, it has been puzzling that the MeCP2 and MBD2 knockouts have given viable, mild phenotypes [26], [30]. Adding to the mystery is the fact that until now, only one endogenous gene has been shown to be affected by a methylated DNA binding protein or activity, and in this case the gene was silenced by the absence of the binding protein, MBD2 [31], the reverse effect.

## Materials and Methods

### Cloning and genomic structure

We used the 3′ UTR sequence of an existing mp66α clone to identify additional mp66α clones in the NCBI mouse EST database, of which only two extended beyond the UTR (AI507147; AI429910). We sequenced these and assembled a sequence (Figure 2) that extended 61 amino acids N terminal of the published *Xenopus* p66α start site [32]. Incomplete genomic sequence in the NCBI database indicated that there was another exon 37 kb upstream that included the probable start site with an imperfect Kozak sequence, but there were gaps in the intervening cDNA sequence. This position, 141 amino acids N terminal of the xp66α start site has now been established as the hp66α start site [17]. Recent mouse ESTs (BY282871; CA317502) place the orthologous start site at 139 amino acids N terminal of the mp66α start site. We will assume this is the mouse start site as the human and mouse genes are almost identical in all other respects.

We probed a BAC library (CITB Mouse BAC Clones, Research Genetics, Cat. No. 96022) derived from mouse strain 129/Sv and obtained 4 positive clones for mp66α (95E12; 412G8; 504L10; 604M1). Except for the 37 kb intron, we mapped the genomic structure using long-range PCR with primers made to the cDNA at regular intervals, and sequenced the amplimers into exon/intron boundaries when introns were detected. We identified critical sites for cloning and verified their presence in the 129/Sv genomic DNA we was using for the construct. For the long-range PCR we used the level 2 protocol of the Expand^TM^ Long Template PCR System (Boehringer Mannheim) but scaled down to a 25 µL reaction volume.

### Gene targeting, ES cell culture, and genotyping

We made the targeting construct by cutting a 1.4 kb short homologous arm and a 6.3 kb long homologous arm from the 129/Sv BAC clone 95E12, then used a shuttle vector to place these arms into the targeting vector, ploxPNT. The targeting vector was designed to replace exons 2–4 of mp66α with a floxed pgkneo cassette. The homologous arms for the targeting construct were cut from BAC 95E12, placed into Bluescript SK-, and finally into the targeting vector ploxPNT (Figure 3) [33]. A portion of each BAC digest was analyzed by Southern blot for identification of the correct band. The HpaII/PstI 1.4 kb short arm fragment was placed into PstI/ClaI site of BS SK- modified by the removal of the Xho1 site by SalI/XhoI digestion and religation. The BamHI/KpnI fragment was then removed and placed into the BamHI/KpnI site of ploxPNT. The EcoRI/DraI 6.3 kb long arm fragment was placed into the EcoRI/SmaI site of BS SK-. The XhoI/NotI fragment was then removed and placed into the XhoI/NotI site of ploxPNT. Internal probes and PCR assays for the short and long arms utilized the following primers: short arm: 2-24 (5′-gcccaccatgccactcttctt-3′), 2-25 (5′-gtcagatgcacaggtccacaac-3′); long arm: 2-14 (5′-cgaggtgggcagcaggtgtct-3′), 2-M2 (5′-ggggacattggcgacacggataag-3′). All junctions of the completed targeting construct were sequenced to confirm correct construction.

Gene targeting was performed on passage #15 R1 ES cells purchased from the Stanford Transgenic Research Facility. This line was developed by the Andras Nagy lab (http://www.mshri.on.ca/nagy/) in 1991 from 129/Svx129/SV-CP stock and has agouti coat color in the F1 generation. ES cells were grown in gelatinized dishes on neomycin resistant γ irradiated mouse embryonic fibroblasts in ES complete medium consisting of DME-H21 (UCSF#AA400) supplemented with 20% fetal bovine serum (Hyclone, ES tested lot AKH12368), 0.1 mM non-essential amino acids (Gibco#11140-0500), 1× penicillin-streptomycin (Gibco #15140-122), 1 mM sodium pyruvate (Gibco#11360-070), 100 µM β-Mercaptoethanol (Sigma # M-7522), and 2000 U/mL murine LIF (ESGRO^TM^, Chemicon ESG1106) (Stanford Transgenic Facility protocol) [34]. The targeting vector was linearized at the Not 1 site and the ES cells were transfected by electroporation. After 24 hours 0.5 mg/mL G418 (Cellgro MT61-234-RF) and 2 µM gancyclovir (Syntex) were added for positive and negative selection. After 7–10 days positive colonies were picked and grown for stocks and analysis. Homologous recombinants were identified first by detection of a 2069 bp band by long range PCR (above) over the short (5′) arm with primers 2-36 (5′-acctggaattgaagctgtctgat-3′) and N-6 (5′-cgccttcttgacgagttcttctg-3′), and then they were confirmed by Southern blot analysis of both arms. For the short arm, ES cell DNA was digested with NcoI and the blots probed with probe 11, a 550 bp fragment amplified from BAC 95E12 with primers 2-39 (5′-tggaaggcacagttaaagggtcac-3′) and 2-40 (5′-ccccgaggtcaggtttcaaggtg-3′). The wild type allele gave a 2.34 kb band while homologously recombined alleles gave a 2.80 kb band. For the long arm, DNA was digested with SpeI and SacI, then probed with probe 9, a 535 bp fragment amplified from BAC 95E12 with primers 2-41 (5′-ggtagcatcccatagtcccaggtt-3′) and 2-42 (5′-atgtggctgattgggggatgg-3′). The wild type gene gave an 8.72 kb band, while homologous recombinants gave a 7.63 kb band (Figure 4).

After southern blot confirmation of homologous recombination in cell and animal lines, a PCR genotyping protocol was developed utilizing primers 2-30 (5′-agacttgagtgtgcagggttgagg-3′), 2-32 (5′-gccgggtattcgagtggaggaggt-3′), 2-Q (5′-aatcacatcaggcgagggaggtct-3′), and N-5 (5′-ttggctacccgtgatattgctgaa-3′). N-5 is found only in the pgk-neo cassette while 2-Q is only in the targeted region; primers 2-30 and 2-32 flank the targeted region. A 25 µL PCR reaction consisting of 0.4 µM primer 2-30, 0.2 µM each remaining primer, 0.8 mM dNTPs, 1.5 U Taq DNA polymerase (Promega, Cat.M2861), 1× Mg-free supplied PCR buffer, 4.5 mM MgCl_2_, 1 µL template, and H_2_O to volume, was cycled as follows: 1 cycle: 94°C, 2 min; 30 cycles: 94°C, 1 min, 61°C, 1 min, 72°C, 1 min; 1 cycle: 72°C, 5 min. Primers 2-30 and N-5 gave an 835 mutant band; primers 2-30 and 2-Q gave a 632 bp wild type band; primers 2-30 and 2-32 gave a 322 bp Δ mutant band and a 2000 bp wild type band.

### Generation and breeding of mice

Correctly targeted ES cells were injected into C57BL/6 blastyocysts [20] at the Stanford Transgenic Research Facility. Chimeric pups were identified by mixed black and agouti coat color and were crossed with C57BL/6 females at 6 weeks of age. The chimeras with the highest percentage of 129/Sv agouti color all developed teratocarcinomas (3) or were sterile (3). Three more were non-transmitters, and three were female. Agouti pup tail DNA was genotyped initially by Southern blot (above) but once the mutation was confirmed in both lines 110 and 26, a PCR protocol was used. The phenotype was analyzed for the most part on a mixed C57BL/6X129/Sv background in which there was no more than 75% of one of the strains. However, we did obtain a few 100% 129/Sv pups for both lines by crossing the proven chimeras with 129/Sv females.

We wanted to eliminate possible confounding effects of the pgk promoter on neighboring genes. To do this, we crossed the heterozygous mp66α females to a CMVCre recombinase male to produce offspring mosaic for the pgkneo deletion. These were then crossed to wild type animals to obtain pure Δpgk mp66α lines for both lines 26 and 110 (Figure 4D). These results were on animals with a mixed 129/Sv X C57Bl/6 background. It was possible that variability would be reduced on an isogenic background. We crossed the chimeras with 129/Sv females and obtained 100% 129/Sv heterozygotes. Although fertility was poor in these lines, we were able to confirm the phenotype in a few (6) embryos on the 100% background. In this case the variability ranged from abnormal looking neural folds at e8.5 to embryos that did not turn at e9.5.

To remove the floxed pgk-neo cassette, we crossed heterozygous mp66α females of both lines, 110 and 26, to a CMV-Cre male on a C57BL/129/SV background. Offspring receiving the CMV-Cre transgene were mosaic for the deletion so these were crossed again to wild type to produce pure Δmp66α lines on a mixed background. The Δmp66α110 and Δmp66α26 lines were then maintained in the heterozygous condition on a mixed background as stock.

### Microarray analysis

Because aberrant RNA is produced in the null animals, we could genotype the embryonic RNA using RT-PCR. Individual RNA from three litters was examined and genotyped for quality and mp66α genotype. Extraembryonic membranes were included with each embryo. Four sibling pairs were chosen from the three litters for microarray analysis. An Affymetrix protocol was used so that each embryo produced an individual set of expression data that could be compared as desired, and all sample pairs were prepared simultaneously. Sex was determined by examining the expression data. Two pairs were entirely female while two other pairs were mixed. The data for each pair was examined in parallel for each gene and genes were not listed unless they were above a for at least three of the four pairs. There was one male each of null and wild type, so these were compared to each other also. The wild type male and wild type females were compared to confirm known male/female differences and to include them in interpretation. In all cases examined for known genes, the results were sex appropriate.

PolyA-RNA was then isolated using the spin column protocol of the Oligotex mRNA midi kit (Qiagen). For microarray analysis, the RNA was quantitated in a Beckman DU530 spectrophotometer and labeled cDNA was made using the Cyscribe labeling kit. The lab reference RNA, (1/4 four-week whole brain : 3/4 e 12.5 complete embryo), was labeled with Cy3 and the ES cell RNA was labeled with Cy5. 0.25 M NaOH was added to degrade RNA during a 15 minute 37°C incubation, then 0.7 M HEPES free acid was added to neutralize. Final purification was carried out with the CyScribe GFX Purification Kit. Absorbance at 550 nm(Cy3) and 650 nm (Cy5) was determined to quantitate incorporation of label, so that equimolar amounts of Cy3 and Cy5 could be used for hybridization. Mouse microarrays were purchased from the SFGF facility, and were stabilized, immobilized, and pre-hybridized as follows. Slides were etched in one corner to identify side of slide printed with the array, and then rehydrated, array side down, over 45°C 1× SSC, then snap-dried on a 100°C surface until the vapor disappeared, then UV cross-linked at 65 mJoules. To prehybridize, arrays were incubated with 50% formamide, 3× SSC, 0.1%SDS, 0.1 mg/mL BSA for 45 minutes at 42°C, immersed in milliQ water to remove SDS, and dried by centrifugation at 600 rpm for 2 minutes.

For hybridization, 40 pmol of reference and ES cell DNA were mixed, 20 ug each of mouse Cot1 DNA, polyA RNA, and tRNA were added, and the sample was concentrated to 28 µL in a foil covered speed-vac. 6 µL 20× SSC and 0.5 µL 20% SDS were added, samples were denatured at 100°C for 2 minutes, and then spun at 14,000 rpm in a microfuge for 15 minutes. The mixture was then pipetted onto the microarray slide and covered. Hybridization was carried out in a Gene Machines HybChamber^TM^ humidified with 3× SSC and submersed in a 65°C water bath for 14–18 hours. The slides were then washed in foil covered slide dishes as follows: 2× SSC, 0.2% SDS at 55°C, 10 minutes, removing cover slips when immersed; 1× SSC. 0.2% SDS at 55°C, 10 minutes; 0.1× SSC, 0.2% SDS at RT, 10 minutes; 0.1× SSC at RT, 10 minutes; distilled water for 10 seconds followed immediately by centrifugation drying as above. The slides were then immediately scanned with a GenePix scanner with photomultiplier tube voltages in each channel adjusted to a ratio of between 0.8–1.2. Images were then gridded and flagged using the GenePix Pro 4.0 Software, then sent to the Stanford Microarray Database.
